# Transcriptome-Based Dissection of the Molecular Mechanisms Underlying Flooding Stress Responses of Eastern Cottonwood in the Floodplains of the Middle and Lower Reaches of the Yangtze River

**DOI:** 10.3390/plants15060958

**Published:** 2026-03-20

**Authors:** Guowei Huang, Xueli Zhang, Xinye Zhang, Ning Liu, Changjun Ding, Jinhua Li, Fenfen Liu, Kailian Long, Chengcheng Gao, Jimeng Sun, Chenggong Liu, Qinjun Huang

**Affiliations:** 1 State Key Laboratory of Tree Genetics and Breeding, Research Institute of Forestry, Chinese Academy of Forestry, Beijing 100091, China; huangguowei1987@163.com (G.H.); zxl961109@163.com (X.Z.); poplarning@caf.ac.cn (N.L.); changjunding@caf.ac.cn (C.D.); lijinh@caf.ac.cn (J.L.); fen@caf.ac.cn (F.L.); gaocc@caf.ac.cn (C.G.); sjm376131292@163.com (J.S.); 2 Research Institute of Forestry (Under-Forest Economy Research Center), Hubei Academy of Forestry, Wuhan 430075, China; ydyxy73@aliyun.com; 3 Key Laboratory of Tree Breeding and Cultivation, State Forestry and Grassland Administration, Beijing 100091, China; 4 Shishou Poplar Research Institute, Hubei Academy of Forestry, Shishou 434400, China; longkailian@126.com

**Keywords:** flooding, abiotic stress, *Populus deltoides*, survival rate, differentially expressed genes, flavonoid biosynthesis

## Abstract

Flooding, as a major abiotic stress, significantly impacts the growth and survival of poplar plantations in the floodplains of the middle and lower reaches of the Yangtze River. Elucidating the molecular mechanisms underlying flooding responses in poplar is crucial for enhancing plantation productivity. In this study, two important eastern cottonwood cultivars, *Populus deltoides* ‘Jianghan 1’ (HBI) and *P. deltoides* Bartr. CL (CL), were investigated. By integrating long-term growth surveys and transcriptome sequencing, we analyzed their phenotypic traits and molecular responses to flooding stress. After 7 years of seasonal flooding, HBI exhibited a survival rate of 73.91%, along with superior height (23.1 m) and diameter at breast height (DBH, 26.3 cm), compared with CL, indicating HBI as a flooding-tolerant cultivar. Transcriptome analysis identified 1098 shared differentially expressed genes (DEGs) in the leaves of flooded HBI and CL, which were mainly enriched in stress signal perception, oxidative stress regulation, energy metabolism and circadian rhythm. Cultivar-specific DEG analysis revealed that CL mainly activated pathways related to oxidative stress and damage repair pathways, whereas HBI-specific genes were significantly enriched in hormone signal transduction, growth regulation, flavonoid synthesis and photosynthesis. Based on this distinct enrichment pattern in the tolerant cultivar HBI, we propose that it possesses adaptive advantages under flooding stress. Specifically, HBI likely coordinates multiple physiological processes by activating ethylene and other hormone-related genes, thereby regulating hypoxia adaptation, reoxygenation-induced oxidative stress, photosynthetic recovery, and flavonoid-mediated antioxidant defense. This coordinated regulation collectively sustains growth vigor and enhances survival under seasonal inundation. Our findings demonstrate clear transcriptomic divergence underlying flooding tolerance among poplar cultivars, laying a theoretical foundation for the selection of flooding-tolerant varieties and the sustainable development of forestry in flood-prone regions. Furthermore, these results broaden the current knowledge of flooding stress biology in woody plants.

## 1. Introduction

In the context of intensifying global climate change, the increasing frequency of extreme weather events—especially heavy precipitation and severe flooding—poses significant threats to plant growth and terrestrial ecosystems [1,2]. Among these threats, hypoxic stress induced by flooding has emerged as one of the most critical abiotic stresses constraining plant growth and development [3,4]. Statistics indicate that flooding causes annual global crop yield losses ranging from 15% to 80%, which largely depend on flooding duration, soil type, and plant species [5,6]. Therefore, a comprehensive understanding of the molecular regulatory mechanisms underlying plant adaptation to hypoxia under flooding condition is of considerable theoretical and practical significance for elucidating plant environmental adaptability and developmental plasticity, as well as for enhancing plant survival following flooding stress [3,7].

Under prolonged flooding or hypoxic stress, the diffusion rates of O_2_ and CO_2_ in water are drastically reduced, while light intensity is weakened and ethylene (ETH) accumulates rapidly, resulting in the inhibition of plant photosynthesis and respiration [8]. Particularly in plant organs or tissues with high oxygen demand (e.g., root tips, leaves, seeds, and fruits), hypoxia acts not only as a stressor but also as a signaling molecule that modulates plant metabolism and developmental processes [9,10]. To cope with this adverse condition, plants have evolved two major adaptive strategies: the quiescence strategy (temporarily suspending metabolism and growth to conserve energy during submergence) and the escape strategy (accelerating organ elongation to reach atmospheric O_2_ above the water surface) [11]. To date, plant responses to flooding have been extensively characterized in numerous crops and various model species [12,13]. For example, plants enhance their flooding tolerance by mobilizing carbohydrates, scavenging reactive oxygen species (ROS), catabolizing sucrose, and selectively translating key mRNAs [14,15,16]. Furthermore, the specific adaptive strategies adopted by plants vary significantly among species, primarily due to inherent genetic differences [5,17]. Flood-tolerant plants (e.g., *Corchorus capsularis* and *Hordeum vulgare*) typically improve their stress resistance by rapidly activating antioxidant systems and reinforcing cell wall biosynthesis [18]. In contrast, *Arabidopsis thaliana* [19] and *Cucumis sativus* [20,21] exhibit enhanced ROS-scavenging capacity and an increased ability to form adventitious roots. Morphologically, plants may also alleviate oxidative damage by suppressing stomatal development, maintaining low levels of cutin and wax biosynthesis, and restricting vascular tissue formation [22].

Beyond interspecific genetic diversity, tissue-specific differences in hypoxic stress responses are also well-documented. As the primary organs directly exposed to hypoxic environments, roots are the first to perceive oxygen deprivation [5,23]. Although aerial tissues are not directly submerged, secondary stresses triggered by root hypoxia lead to reduced chlorophyll content, impaired photosynthetic efficiency, and decreased stomatal conductance, thereby accelerating leaf senescence and altering resource allocation [24,25]. In recent years, multiple signaling pathways associated with plant survival under submergence have been progressively elucidated. These include hypoxia signaling, energy transduction signaling, ETH signaling—where the key transcription factor EIN3 activates cell elongation-related genes to promote escape growth under flooding conditions [26]—and metabolite synthesis signaling (e.g., up-regulate flavonoid biosynthesis genes in aerial tissues to mitigate reactive oxygen species (ROS) damage [27], ROS signaling, and mitochondrial signaling [28,29,30,31]). Research in this field has also expanded to incorporate multi-omics approaches, transcription factor characterization, and gene editing technologies [32,33,34]. Collectively, these studies demonstrate that flooding tolerance arises from the synergistic interactions of morphological structures, physiological and biochemical processes, and molecular regulatory mechanisms. However, the molecular mechanisms governing flooding responses in woody plants—particularly fast-growing timber species such as poplars—remain far less characterized compared to those in herbaceous crops. This knowledge gap is primarily attributed to the perennial growth habit and complex genome architecture of woody plants.

The floodplains in the middle and lower reaches of China’s Yangtze River are seasonally inundated wetlands characterized by flat terrains, covering a total area of approximately 6.3 × 10^5^ hm^2^. Owing to annual heavy rains and flooding during the wet season, this region exhibits a distinct climate pattern of dry winters and wet summers, coupled with diverse wetland types and unique seasonal inundation characteristics [35,36]. Furthermore, influenced by the seasonal water level fluctuations of the Yangtze River, the annual inundation period in summer ranges from 0 to 60 days, which severely restricts the selection of suitable plant species [37]. While the silt deposited during flooding is widely recognized to be rich in nutrients, potentially enhancing soil fertility [38], prolonged submergence exerts significant adverse effects on plant growth and yield stability in this region. For example, cotton (*Gossypium hirsutum*) shows a marked reduction in fiber length and strength under such conditions [39]. Poplars are among China’s most vital fast-growing timber species and are extensively cultivated nationwide [40,41]. In particular, *Populus deltoides*, native to the lower Mississippi River basin of North America, has a natural distribution ranging from southern Canada to the southeastern United States [42]. First introduced to China in the 1950s, several superior and well-adapted clones or cultivars of this species have since been developed [43], playing a particularly crucial role in ecological conservation and timber production in the floodplains of the middle and lower Yangtze River [44]. Taking the Lijiazhou Forest Farm in Huanggang City as a case study, the planted *P. deltoides* stands are subjected to annual flooding from June to August, with water depths reaching 2–3 m and inundation durations lasting 1–2 months. Under sustained flooding stress, poplar trees exhibit pronounced morphological abnormalities, including reduced growth rates, leaf chlorosis, wilting, and defoliation. For varieties with low adaptability, root activity often declines sharply, eventually leading to root necrosis [45]. This observation is consistent with our previous stand survey findings, where some poplar varieties gradually died following years of seasonal flooding, whereas others exhibited greater longevity. This differential survival may be attributed to the development of an efficient antioxidant enzyme system in flood-tolerant varieties, which helps regulate physiological processes and maintain viability under flooding stress [46].

Additionally, previous studies have primarily focused on the phenotypic plasticity and adaptive responses of poplars to summer flooding or waterlogging stress during their growth and development stages [47]. For instance, Nielsen et al. [48] and Rood et al. [49] demonstrated that female *P. angustifolia* exhibits greater flood tolerance than male; females are likely more successful in low-lying, flood-prone habitats, with significant intraspecific differences also observed [46,50]. However, the molecular mechanisms underlying these stress responses and their variations remain poorly elucidated. Therefore, based on the long-term investigation of perennial experimental forests conducted in previous years, this study integrates leaf transcriptome analysis to address the following key objectives: (i) to verify whether interspecific differences exist in the growth performance and survival potential of poplars in response to seasonal flooding stress; (ii) to determine whether the responses of different poplar varieties to flooding stress are associated with the differential expression of specific genes; and (iii) to explore whether the adaptive advantage of flood-tolerant poplars in coping with flooding stress is linked to ETH and flavonoid biosynthesis pathways. The research findings will theoretically clarify the differential adaptation mechanisms among flood-tolerant poplar varieties, provide crucial theoretical support for the breeding of flood-tolerant poplars, and expand the research frontier of flood stress tolerance and molecular stress resistance in forest trees.

## 2. Results

### 2.1. Effects of Flooding on Growth and Survival of Populus deltoides

**Figure 1:**
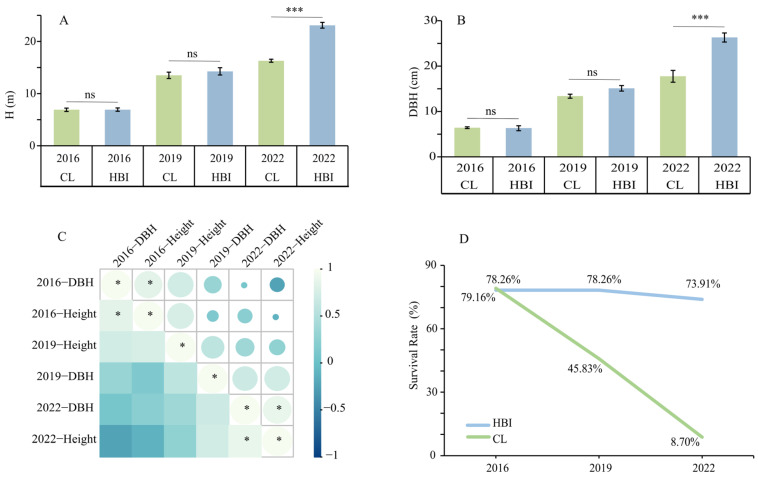
Growth performance and survival of the eastern cottonwood cultivars HBI and CL across different years. (**A**,**B**) Changes in tree height (H) and diameter at breast height (DBH) from 2016 to 2022. (**C**) Correlation analysis between DBH and H. (**D**) Trends in survival rate. ns, not significant; *** *p* < 0.001; * *p* < 0.05.

Figure 1 shows that the two eastern cottonwood cultivars, HBI and CL, exhibited clear differences in growth performance and survival under long-term seasonal flooding. During the period from 2016 to 2022, both diameter at breast height (DBH) and tree height (H) increased with stand age for both cultivars, with HBI consistently exhibiting greater growth increment than CL. By 2022, the mean H (23.1 m) and DBH (26.3 cm) of HBI were 41.72% and 48.17% higher than those of CL, respectively (Figure 1A,B). Correlation analysis revealed a positive relationship between DBH and H within the same year, with significant positive correlations observed in 2016 and 2022 (*p* < 0.05). This indicates coordinated growth between height and radial increment (Figure 1C). Regarding survival, HBI maintained a relatively high survival rate from 2016 to 2022 (78.26–73.91%), whereas CL’s survival rate declined sharply from 79.16% to only 8.70% by 2022 (Figure 1D). These results indicate that long-term flooding exerted a significantly stronger inhibitory effect on CL, while HBI displayed superior flooding tolerance and growth performance—confirming its classification as a flooding-tolerant cultivar.

### 2.2. Quality Assessment of RNA-Seq Data

The quality evaluation of the transcriptome sequencing data is summarized in Table 1. The proportion of clean reads exceeded 98.20% across all samples. After quality control, the amount of clean data ranged from 5.39 to 7.94 Gp, with an average of 6.47 Gp. The overall mapping rate was high (96.45–98.05%), indicating excellent alignment between the sequencing reads and the reference genome. In addition, Q20 values were above 98.27% for all samples, while Q30 values ranged from 92.94% to 97.20%, demonstrating high base-calling accuracy. The GC content was relatively stable (43.71–44.23%), suggesting no obvious compositional bias among samples. The proportion of ambiguous bases (N) was lower than 0.0053% in all libraries, further confirming the reliability of the sequencing data.

**Figure 2:**
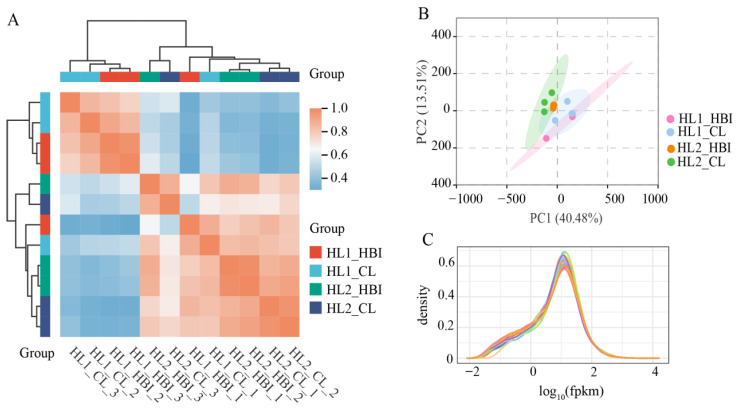
Quality assessment of the transcriptome data. (**A**) Sample correlation analysis. (**B**) Principal component analysis (PCA). (**C**) Density distribution of gene expression levels. HL1, before flooding; HL2, after flooding.

Sample correlation analysis and principal component analysis (PCA) indicated that the flooding treatment (HL2 vs. HL1) was the primary factor driving transcriptomic variation in the leaves of both cultivars (Figure 2A,B). Moreover, density distributions of gene expression levels across samples showed highly overlapping normal curves (Figure 2C), indicating comparable global expression patterns among treatments. Together, these results demonstrate that the sequencing datasets are of high quality and meet the requirements for subsequent differential expression analysis, functional annotation, and pathway enrichment, thereby supporting downstream bioinformatic investigations.

### 2.3. DEGs in HBI and CL Before and After Flooding

**Figure 3:**
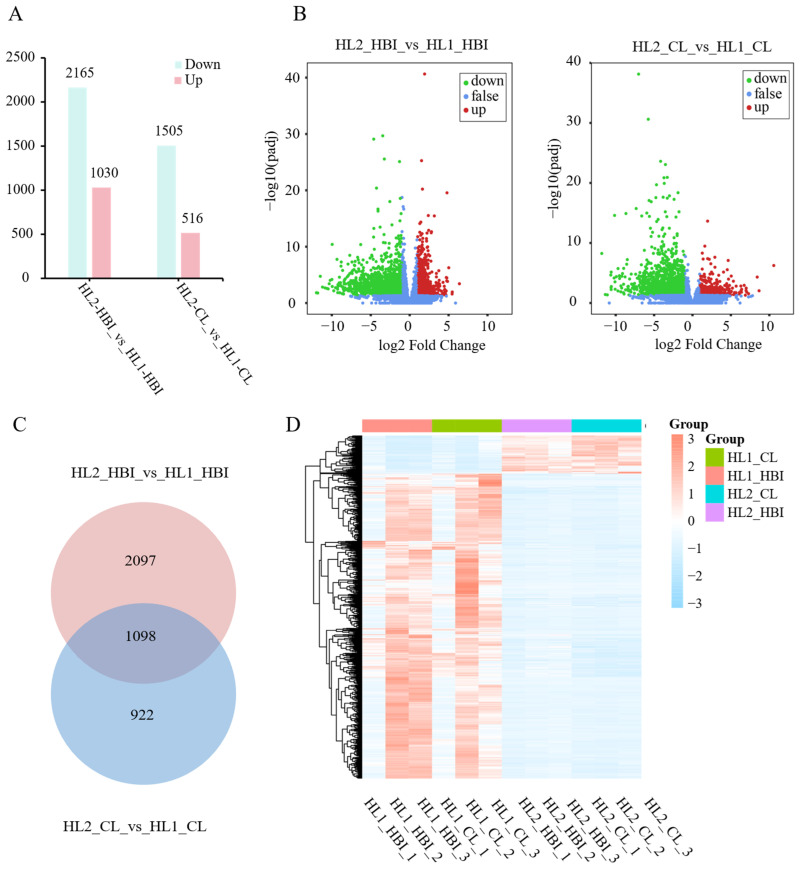
Identification of DEGs and their expression patterns in leaves of HBI and CL before and after flooding. (**A**) Statistics of up- and down-regulated DEGs. Blue bars represent the number of down-regulated genes, and pink bars represent the number of up-regulated genes. (**B**) Volcano plot of DEGs. (**C**) Venn diagram showing shared and cultivar-specific DEGs. (**D**) Heatmap of expression patterns of the common DEGs.

Following flooding stress, a total of 3195 differentially expressed genes (DEGs) were identified in the leaves of HBI, including 1030 up-regulated and 2165 down-regulated genes. In contrast, 2021 DEGs were detected in CL, with 516 up-regulated and 1505 down-regulated. In both cultivars, the number of down-regulated genes was markedly higher than that of up-regulated genes (Figure 3A,B). A comparison between the two cultivars revealed 1098 DEGs shared by HBI and CL after flooding treatment (Figure 3C), and these common genes exhibited similar expression patterns in both cultivars. Among the shared DEGs, 971 were down-regulated and 127 were up-regulated, with the number of down-regulated genes being approximately 7.6 times greater than that of up-regulated genes (Figure 3D). In addition, 2097 cultivar-specific DEGs were identified in HBI, while 922 were unique to CL.

**Figure 4:**
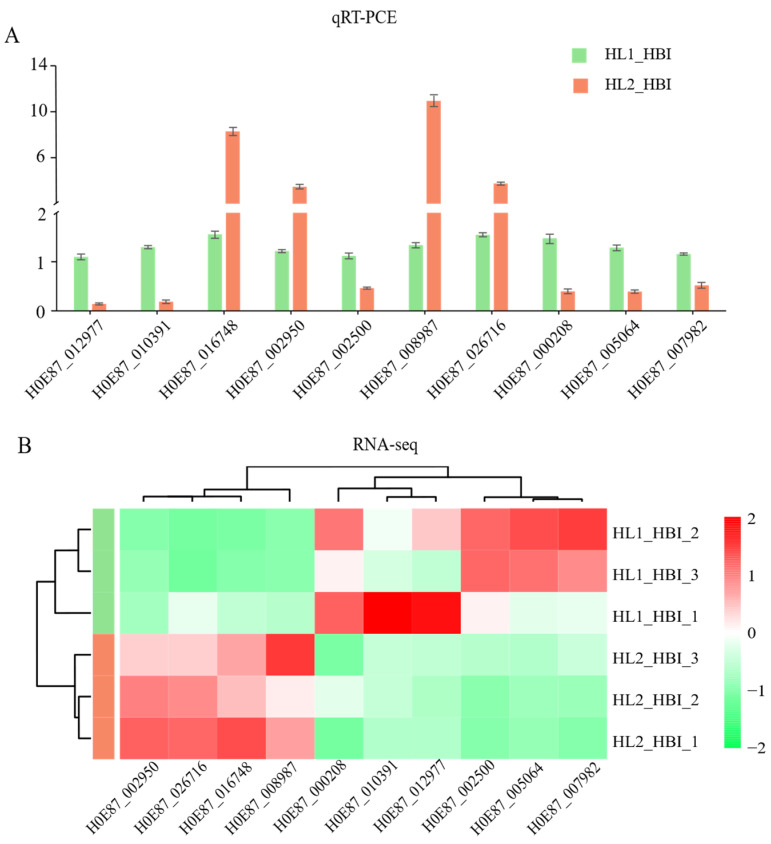
Validation of RNA-seq results by qRT-PCR. (**A**) qRT-PCR analysis of ten selected genes. (**B**) Corresponding expression patterns derived from the transcriptome data.

To validate the reliability of the RNA-seq results, ten genes specifically expressed in the flooding-tolerant cultivar HBI were randomly selected for quantitative real-time PCR (qRT-PCR) analysis. Their expression levels under flooding treatment were examined and compared with the corresponding transcriptome data. The qRT-PCR results showed that four genes (*H0E87_016748*, *H0E87_002950*, *H0E87_008987* and *H0E87_026716*) were significantly up-regulated, while the other six were significantly down-regulated under flooding stress (Figure 4A)—a pattern highly consistent with the RNA-seq data (Figure 4B). These findings confirm the reliability and accuracy of the transcriptome analysis in this study.

### 2.4. Functional Annotation of Shared DEGs in HBI and CL Before and After Flooding

**Figure 5:**
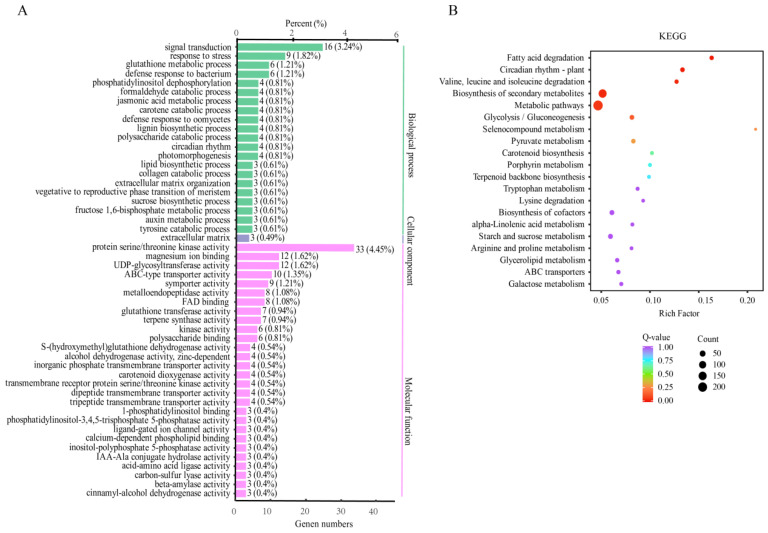
GO annotation and KEGG pathway enrichment of the common differentially expressed genes (DEGs) in leaves of HBI and CL. (**A**) GO enrichment analysis. (**B**) KEGG pathway enrichment.

To elucidate the shared molecular responses of the flooding-tolerant cultivar HBI and the flooding-sensitive cultivar CL, Gene Ontology (GO) and KEGG pathway enrichment analyses were performed on the DEGs shared by both cultivars. GO enrichment analysis revealed that these common DEGs were significantly overrepresented in biological processes, including signal transduction (16 DEGs), response to stress (9 DEGs), glutathione metabolic process (6 DEGs), and defense response to bacterium (6 DEGs). In the molecular function category, the common DEGs were mainly enriched in protein serine/threonine kinase activity (33 DEGs), magnesium ion binding (12 DEGs), UDP-glycosyltransferase activity (12 DEGs), ABC-type transporter activity (10 DEGs), and symporter activity (9 DEGs) (Figure 5A).

KEGG pathway analysis further indicated that the shared DEGs were primarily involved in fatty acid degradation (16 DEGs), circadian rhythm—plant (20 DEGs), valine, leucine and isoleucine degradation (14 DEGs), biosynthesis of secondary metabolites (148 DEGs), and general metabolic pathways (233 DEGs) (Figure 5B). These results suggest that HBI and CL may employ common mechanisms, including stress perception, defense response, metabolic reprogramming, and circadian coordination, to cope with flooding stress.

### 2.5. Functional Annotation of Cultivar-Specific DEGs in HBI and CL

**Figure 6:**
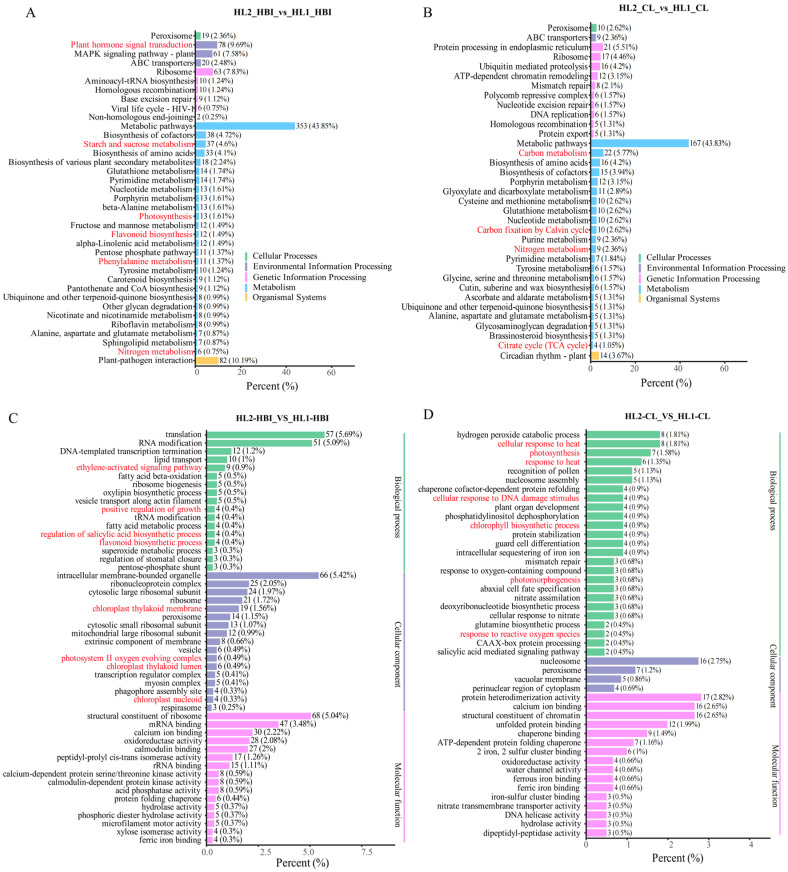
GO annotation and KEGG pathway enrichment of cultivar-specific differentially expressed genes (DEGs) in leaves of HBI and CL before and after flooding. (**A**,**B**) KEGG pathway enrichment of HBI- and CL-specific DEGs, respectively. (**C**,**D**) GO enrichment of HBI- and CL-specific DEGs, respectively.

KEGG enrichment analysis of cultivar-specific DEGs under flooding stress revealed distinct differences between HBI (flood-tolerant) and CL (flood-sensitive). In HBI, the unique DEGs were significantly enriched in pathways including plant hormone signal transduction, MAPK signaling pathway, starch and sucrose metabolism, photosynthesis, flavonoid biosynthesis, and phenylalanine metabolism (Figure 6A). In contrast, CL-specific DEGs were mainly enriched in photosynthesis-related pathways, such as photosynthesis, chlorophyll biosynthetic process, and photomorphogenesis (Figure 6B).

GO enrichment analysis provided further insights into the divergent response patterns. In HBI, the unique DEGs were primarily involved in biological processes, including the ethylene-activated signaling pathway, positive regulation of growth, and flavonoid biosynthetic process. At the cellular component level, these DEGs were significantly enriched in the photosystem II oxygen-evolving complex and chloroplast (Figure 6C). These findings are consistent with the KEGG results (Figure 6A), further suggesting that hormone signaling (particularly ethylene), MAPK cascades, flavonoid metabolism, and photosynthetic regulation may play pivotal roles in HBI’s response to flooding stress.

By comparison, CL-specific DEGs were enriched in hydrogen peroxide catabolic process, cellular response to DNA damage stimulus, response to oxygen-containing compounds, and response to reactive oxygen species (Figure 6D). This pattern indicates that under flooding stress, CL may predominantly activate oxidative stress and damage repair pathways, accompanied by substantial alterations in the expression of photosynthesis-related genes.

### 2.6. Flavonoid Biosynthesis Pathway Involved in the Flooding Response of Populus deltoides

To further elucidate the regulatory mechanisms underlying the enrichment of flavonoid biosynthesis in the flooding-tolerant cultivar HBI, a total of 12 significantly enriched DEGs were identified in this pathway. Among these, three genes (*HOE87_019508*, *HOE87_020833*, and *HOE87_016748*) were up-regulated under flooding stress, whereas the remaining nine were down-regulated.

**Figure 7:**
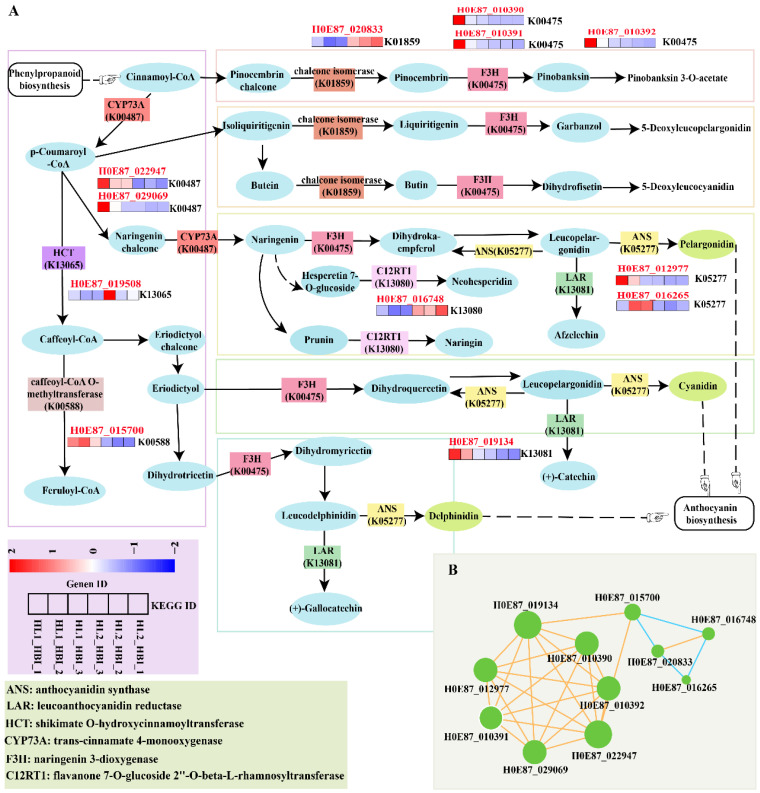
Flavonoid biosynthesis network in leaves of the flooding-tolerant cultivar HBI under flooding stress. (**A**) Schematic diagram of the flavonoid biosynthetic pathway and expression profiles of the 12 associated DEGs. Ellipses indicate metabolic intermediates, boxes represent key enzymes enriched among the DEGs, and the heatmap shows the corresponding gene expression patterns. (**B**) Correlation network of genes involved in the flavonoid biosynthesis pathway.

These genes are primarily involved in key steps of the flavonoid biosynthetic pathway, particularly the catalytic processes governing anthocyanin production. They include genes encoding anthocyanidin synthase (ANS), leucoanthocyanidin reductase (LAR), flavanone 3-hydroxylase (F3H), chalcone isomerase (CHI), caffeoyl-CoA O-methyltransferase (CCoAOMT), hydroxycinnamoyl-CoA shikimate/quinate hydroxycinnamoyltransferase (HCT), and trans-cinnamate 4-monooxygenase (C4H/CYP73A) (Figure 7A).

The differential expression of these enzymes suggests a specific regulatory adjustment of flavonoid accumulation and anthocyanin biosynthesis in HBI under flooding conditions. Furthermore, correlation analysis revealed significant transcriptional associations among 11 DEGs within the flavonoid biosynthesis pathway (Figure 7B), indicating coordinated transcriptional regulation of this pathway in response to flooding stress.

### 2.7. Distinct Response Patterns of HBI and CL Under Flooding Stress

**Figure 8:**
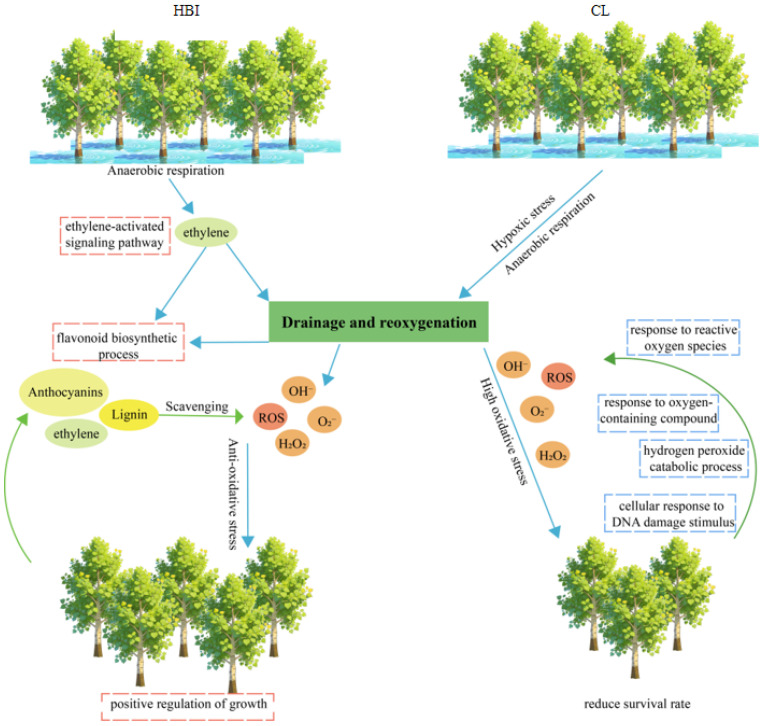
Schematic diagram of regulatory mechanisms in HBI and CL under flooding stress. Under flooding stress, both the *P. deltoides* varieties HBI and CL are exposed to hypoxic stress. The flood-tolerant cultivar HBI can specifically activate ethylene and other hormone-related genes, thereby constructing a synergistic regulatory network associated with flavonoid biosynthesis. This network modulates and scavenges the accumulation of harmful ROS in the plants during flooding stress and subsequent drainage (reoxygenation), and positively regulates the growth and development of the poplars. In contrast, the flood-sensitive cultivar CL does not exhibit such an ethylene–flavonoid regulatory network. Instead, it responds to ROS accumulation via pathways including the response to reactive oxygen species, response to oxygen-containing compounds, hydrogen peroxide catabolic process, and cellular response to DNA damage stimulus.

The contrasting DEG enrichment patterns between HBI and CL suggest that the flooding-tolerant cultivar HBI may employ an intrinsic adaptive strategy distinct from that of CL. Based on these findings, we propose a regulatory model illustrating the adaptive advantage of HBI under flooding stress (Figure 8). In this model, HBI is hypothesized to establish a coordinated multi-pathway regulatory network via the specific activation of ethylene and other hormone-related genes. This network is presumed to simultaneously orchestrate hypoxic adaptation, defense against reoxygenation-induced oxidative stress, the recovery of photosynthetic function, and flavonoid-mediated antioxidant defense. Through such integrated regulatory mechanisms, HBI is able to maintain growth vigor and enhance survival in seasonally flooded environments.

## 3. Discussion

Among abiotic stresses, flooding is widely recognized as one of the most critical factors affecting the growth, geographical distribution, productivity, and survival of vegetation worldwide. It primarily impairs plant development by inducing hypoxic stress, ion toxicity, and energy deficiency [4,10,51]. Studies have shown that under flooding stress, a range of plant phenotypic and physiological traits—including plant height, stem diameter, photosynthetic parameters, and chlorophyll content—are significantly suppressed [52], and flood tolerance varies considerably among genotypes with distinct genetic backgrounds [5,53]. In this study, the survival rates of the *P. deltoides* cultivars HBI and CL decreased progressively with increasing flooding duration, which is consistent with the findings of Sun et al. [54]. This result indicates that prolonged seasonal flooding during the growing season can lead to mortality in poplars. Furthermore, HBI exhibited distinct growth advantages relative to CL and is thus classified as a flood-tolerant poplar cultivar. This finding provides a solid foundation for the genetic enhancement of poplars and the identification of flooding-resistant genes, while further confirming the marked differences in flood tolerance among plant genotypes [17].

It is well established that severe flooding stress compels plants to up-regulate anaerobic respiration in their root systems, which subsequently impairs photosynthesis and aerobic metabolic processes [55]. As a key hormonal regulator of plant responses to flooding, ethylene not only triggers adaptive root reactions but also acts as a long-distance signal to modulate foliar responses in aboveground tissues [56]. Furthermore, ethylene can alleviate oxidative stress and enhance metabolic adaptation by preconditioning plants to hypoxic stress through nitric oxide (NO) consumption, activation of anaerobic pathways, and regulation of ROS metabolism, thereby fortifying plant survival mechanisms prior to the onset of hypoxia [29,55,57]. In this study, we identified 78 cultivar DEGs enriched in the plant hormone signal transduction pathway in the flood-tolerant cultivar HBI. Among these, 10 DEGs (e.g., *H0E87_000208*, *H0E87_018198*, *H0E87_003977*, etc.) were directly associated with the ETH signaling pathway, accounting for 12.82% of the hormone-related DEGs, highlighting the core role of ETH in HBI’s response to flooding stress. In contrast, no significant enrichment of plant hormone signaling pathways was detected in CL. Instead, CL-specific genes were significantly enriched in oxidative stress and damage repair pathways, such as the hydrogen peroxide catabolic process and ROS accumulation pathways. This discrepancy may be attributed to a sudden oxygen burst in CL tissues upon drainage after prolonged hypoxia, which induced ROS accumulation and consequently activated robust oxidative stress and damage repair pathways to maintain ROS homeostasis [55,58]. This observation is consistent with the findings of Yu et al. [59] in *A. thaliana*. However, no differential expression of oxidative stress and damage repair-related genes was observed in the flood-tolerant cultivar HBI. This may be because flooding activated the specific expression of genes associated with ETH and other plant hormones in HBI. As reported in cotton [60,61] and *Arabidopsis* [62], enhanced adaptability to hypoxic stress can be achieved through the dynamic regulation of ETH signaling-related genes during continuous flooding.

Additionally, ETH modulates plant photosynthetic processes during the regulation of hypoxia induced by flooding stress and subsequent reoxygenation [1,55,63]. For instance, the photosynthetic capacity of *Zanthoxylum armatum* seedlings decreased during the reoxygenation phase following flooding, while exogenous ETH application partially mitigated the damage caused by prolonged flooding. However, excessive ETH accumulation has been shown to inhibit overall photosynthetic function [64]. Similar findings have been reported in poplars: studies have demonstrated that exogenous ETH significantly increases the net photosynthetic rate, transpiration rate, and root hydraulic conductivity of *P. tremuloides* under hypoxic conditions [65]. In the present study, flooding stress significantly upregulated the expression of genes involved in multiple photosynthesis-related pathways (e.g., photosynthesis, chlorophyll biosynthetic process, photomorphogenesis) in both HBI and CL. This suggests that both cultivars may counteract flooding-induced photosynthetic inhibition by activating the expression of genes associated with photosynthesis-related pathways. Among these regulatory mechanisms, ETH signaling likely plays a pivotal role in mediating this adaptive response in the flood-tolerant cultivar HBI [55]. Therefore, future research should further utilize genetic manipulation of key ETH synthesis or signaling genes to clarify the specific molecular mechanisms by which ETH signaling regulates oxidative stress responses and photosynthetic recovery during flooding-induced hypoxia and post-drainage reoxygenation.

Previous studies have demonstrated that plants can trigger the synthesis of secondary metabolites under stress conditions, including flavonoids, phenolics, isoflavonoids, flavonols, and anthocyanins [40,66,67,68]. Additionally, ETH promotes the accumulation of flavonols in plant roots and stomatal guard cells [69,70]; specifically, it induces flavonoid biosynthesis in guard cells via the EIN2 signaling pathway. These flavonoids act as potent antioxidants that scavenge ROS, thereby antagonizing the ABA signaling pathway and playing crucial roles in diverse biological processes as well as in defense against abiotic stresses [71]. In the present study, cultivar-specific DEGs in the waterlogging-tolerant genotype HBI were significantly enriched in the flavonoid biosynthesis pathway These included 12 DEGs encoding key enzyme-encoding genes such as *F3H*, *C12RT1*, *CHI*, *C4H*, *HCT*, *ANS*, *LAR*, and *CCoAOMT* (note: *C12RT1* was redundantly mentioned in the original text and has been adjusted to avoid repetition). This finding is consistent with observations in quinoa (*Chenopodium quinoa*) under flooding stress, where coordinated regulation of flavonoid biosynthesis by key enzyme genes (e.g., *CHS*, *F3H*, *FLS*, *CHI*) and transcription factors (e.g., *MYB*, *NF-YC*) enhances antioxidant defense capacity and flooding tolerance [72,73]. Hence, we speculate that the ETH-mediated flavonoid biosynthesis pathway in HBI is activated mainly through the ETH signaling pathway. This pathway regulates the expression of key flavonoid synthesis genes, including *HOE87_020833* (*CHI*) and *HOE87_016748* (*ANS*), thereby establishing an ETH-flavonoid regulatory module. Collectively, these results indicate that the ETH-mediated flavonoid biosynthesis pathway under flooding stress may act as a crucial survival mechanism that enhances flood tolerance in *P. deltoides* HBI.

This study constructed the flavonoid biosynthesis pathway in HBI and analyzed the expression patterns of DEGs significantly enriched in this pathway. The first segment corresponds to the phenylpropanoid metabolic pathway, a common biosynthetic hub for flavonoids, chlorogenic acid, and lignin. Among these metabolites, C4H acts as a key enzyme at the branch point of the flavonoid biosynthesis pathway, catalyzing the formation of *p*-coumaroyl-CoA [74,75,76]. For example, previous studies have demonstrated that specific silencing of the tobacco *NtC4H* gene can upregulate the expression of flavonoid pathway-related genes such as *Nt4CL* and *NtCHS*, thereby significantly enhancing the accumulation of target flavonoid products (e.g., pinostrobin, naringenin) and chlorogenic acid [77]. In the flavonoid biosynthesis pathway, *CHI* and *F3H* are responsible for constructing the basic flavonoid skeleton and determining major biosynthetic branches, serving as core components of flavonoid synthesis and constituting the second segment [78]. In HBI, we identified three specifically down-regulated *F3H* genes (*H0E87_010390*, *H0E87_010391*, *H0E87_010392*) and one up-regulated CHI-related gene (*H0E87_020833*), which encodes as a key enzyme involved in flavanone production. The third segment comprises the specialized downstream branch of flavonoid biosynthesis, which is responsible for the synthesis of specific classes of flavonoid end products. For instance, key enzymes including ANS and LAR are involved in the synthesis of anthocyanins and proanthocyanidins [79], while enzymes such as HCT and CCoAOMT participate in lignin biosynthesis [80,81]. Consistently, during the flooding–reoxygenation process in HBI, we identified DEGs associated with these key enzymes, including *ANS* genes (*H0E87_012977*, *H0E87_016265*), *LAR* (*H0E87_019134*), *HCT* (*H0E87_019508*) and CCoAOMT (*H0E87_015700*).

In recent years, anthocyanins and lignin have garnered considerable attention owing to their prominent physicochemical activities, such as antioxidant properties and ROS scavenging capacity [82,83]. For instance, previous studies have shown that both *F3H* and *ANS* belong to the 2-oxoglutarate/Fe(II)-dependent dioxygenase (2-ODDs) family. Heterologous expression of *Pn2-ODD1* significantly enhanced the tolerance of *Arabidopsis* to salt, drought, UV-B, and oxidative stresses by promoting the synthesis of anthocyanins and flavonols [84]. In the present study, under flooding stress, HBI did not rapidly activate ROS response and damage repair pathways in the same manner as CL. Instead, a specific flavonoid biosynthesis pathway was identified, which indirectly suggests that HBI was not subjected to secondary damage caused by post-drainage reoxygenation stress. Therefore, we speculate that HBI may dynamically regulate the processes of flooding-induced hypoxia and drainage-induced reoxygenation by modulating the expression of genes encoding key enzymes in the flavonoid biosynthesis pathway and scavenging ROS. However, this study only analyzed transcriptome-level data without verifying the actual accumulation of flavonoids and ETH via metabolomics; the root, as the primary organ sensing flooding stress, was excluded from the transcriptome analysis; and functional validation of candidate genes was limited to qRT-PCR without in vivo or field verification. Based on these limitations, future research will integrate metabolomics and proteomics to validate the accumulation of flavonoids and ETH in HBI under flooding stress, analyze root transcriptomes to elucidate early flooding response mechanisms, and perform functional validation of core candidate genes (e.g., *HOE87_018198, HOE87_020833*) through transgenic poplar field experiments. This will lay a solid theoretical foundation for thoroughly elucidating the molecular regulatory mechanisms underlying poplar flooding tolerance and facilitating its genetic improvement.

## 4. Materials and Methods

### 4.1. Plant Materials and Experimental Site

Two cultivars, HBI (*P. deltoides* ‘Jianghan 1’) and CL (*P. deltoides* ‘Chulin 2’), bred by the Hubei Academy of Forestry (E 114°56′, N 30°52′), were used as the experimental materials in this study. HBI is an elite provincial variety of Hubei Province (Certification No. ER-SC-PD-001-2020), developed in 2012 through controlled hybridization between *P. deltoides* ‘Zhongshi 8’ and *P. deltoides* ‘2-46’. CL (Certification No. ER-SC-PD-009-2009) was selected in 2009 from the natural hybrid progeny of *P. deltoides* ‘I-69’. In January 2015, uniform and healthy two-year-old nursery trees with straight stems and no visible pests or diseases were selected for afforestation at the Lijiazhou Forest Farm (114°42′ E, 30°25′ N). The planting density was 3 m × 10 m. Trees were established using a cutback planting method, with an average initial height of 5.2 ± 0.16 m and an average DBH of 3.8 ± 0.18 cm at the time of cutting. Each cultivar was arranged in a randomized block design with three replicates and six trees per plot. In addition to HBI and CL, the trial plantation also included 184 other eastern cottonwood cultivars (e.g., *P. deltoides* ‘Jianghan 3’), covering a total area of 57 mu, with the same afforestation method applied. The experimental site is located within the embankment of the Yangtze River and is characterized by a subtropical monsoon climate. It represents a typical seasonally flooded wetland, where the maximum flood depth during the flood season (June–August) can reach up to 3 m. Detailed information on the plant materials and site conditions is provided in Table 2.

### 4.2. Growth Measurement and Data Analysis

H and DBH of HBI and CL were individually measured after leaf fall in 2016, 2019, and 2022, and survival rates were recorded simultaneously. H was measured using an ultrasonic hypsometer (Vertex 5, Haglöf, Långsele, Sweden) with an accuracy of 0.1 m. DBH was determined using a diameter tape (YM-CL001, Yuma Tools Co., Ltd., Zhengzhou, China) with a precision of 0.01 cm.

Data compilation and preliminary processing were performed using Microsoft Excel (v2019, Microsoft Corp., State of Washington, USA). Descriptive statistics, one-way analysis of variance (ANOVA), and Duncan’s multiple range test were conducted using SPSS (v21.0). Correlation analyses were performed in R (v3.1.2).

### 4.3. RNA Sequencing and Quality Control

Leaf samples were collected from HBI and CL before flooding (29 May 2024) and after water recession (20 August 2024) during the flood season in the middle and lower Yangtze River floodplain. Functional leaves were harvested from current-year shoots at comparable heights and the same orientation. For each cultivar and time point, leaves from three branches were sampled as three single-plant biological replicates. Samples were wrapped in aluminum foil, labeled, and immediately frozen in liquid nitrogen for RNA extraction.

The extraction of RNA was performed using the Trizol Reagent kit (Ambion, Carlsbad, CA, USA), and RNA sequencing was performed by Personalbio Co., Ltd. (Shanghai, China). Raw reads were filtered using fastp (v0.23.4) to remove adapter sequences at the 3′ end and reads with an average quality score below Q20. Q20, Q30, and GC content were subsequently calculated to evaluate sequencing quality.

### 4.4. Gene Expression Quantification and Identification of DEGs

Clean reads were aligned to the *P. deltoides* reference genome (WV94_445_v2.0.fa) using HISAT2 (v2.2.1). Read counts mapped to each gene were calculated with HTSeq-count (v0.13.5) as the raw expression values. Gene expression levels were normalized as FPKM (fragments per kilobase of transcript per million mapped reads).

Differential expression analysis was conducted using DESeq2 in R (v3.5.1). Genes meeting the criteria of |log2FoldChange| > 1 and *p*-value < 0.05 were considered significantly differentially expressed. Bidirectional hierarchical clustering of DEGs and samples was performed using the pheatmap package, and volcano plots were generated with ggplot2.

### 4.5. GO Annotation and KEGG Enrichment Analysis

Gene functions were annotated using the Gene Ontology (GO) database and the Kyoto Encyclopedia of Genes and Genomes (KEGG) database. Enrichment analyses were conducted with clusterProfiler. For each GO term or KEGG pathway, the list of DEGs and the corresponding gene counts were calculated to determine significantly enriched functional categories.

### 4.6. qRT-PCR Validation of DEGs

Ten genes specifically expressed under flooding stress in the tolerant cultivar HBI were randomly selected for validation. Primers were designed based on *P. deltoides* sequences using Primer3web (https://primer3.ut.ee/, accessed on 17 November 2025). RNA was extracted from HBI leaves collected before and after flooding, and gene expression levels were quantified using an Applied Biosystems 7500 Real-Time PCR System (Thermo Fisher Scientific, Waltham, MA, USA). The ubiquitin gene served as the internal reference, with primer sequences ubiquitin_qRT-F: 5′-GTTGATTTTTGCTGGGAAGC-3′ and ubiquitin_qRT-R: 5′-GATCTTGGCCTTCACGTTGT-3′ [86]. Relative expression levels were calculated using the 2^−ΔΔCt^ method [87].

## 5. Conclusions

Flooding-induced hypoxic stress has emerged as a major abiotic stress constraining plant growth and development. In this study, we compared the survival rates, H and DBH of two *P. deltoides* cultivars (HBI and CL) under long-term flooding stress. The results demonstrated that HBI exhibited superior flood tolerance and survival adaptability compared with CL, as evidenced by its greater H (21.3 m), DBH (26.3 cm), and survival rate (73.91%). Transcriptome analysis revealed that the DEGs in the leaves of HBI and CL under flooding stress were primarily annotated to and enriched in pathways related to stress signal perception, oxidative stress regulation, energy metabolism remodeling, photosynthesis, and circadian rhythm coordination. These pathways collectively mitigate the growth and developmental constraints imposed by flooding. Notably, post-flooding, CL likely experienced acute oxidative stress, as indicated by the significant enrichment of its DEGs in pathways such as hydrogen peroxide catabolic process, cellular response to DNA damage stimulus, response to oxygen-containing compounds, and response to ROS. In contrast, the flood-tolerant cultivar HBI specifically activated the expression of 78 unique phytohormone-related genes upon exposure to flooding stress, 10 of which are directly associated with the ETH signaling pathway. These genes collectively and dynamically regulate hypoxia–hyperoxia stress, photosynthetic inhibition, and the accumulation of antioxidant flavonoids, thereby endowing HBI with enhanced adaptive capacity and survival mechanisms. Future research will integrate metabolomic and proteomic analyses to explore and validate relevant functional genes. Additionally, more comprehensive investigations will be conducted on waterlogging-sensitive tissues and organs (e.g., roots) to better elucidate and reveal the molecular regulatory mechanisms underlying *P. deltoides* response to seasonal flooding.

## Figures and Tables

**Table 1:** Summary of transcriptome sequencing data.

Sample	Clean Data (Gp)	Clean Reads (%)	Mapped Rate (%)	GC (%)	N (%)	Q20 (%)	Q30 (%)
HL1_HBI_1	7.94	98.43	97.74%	43.98	0.005298	98.57	94.15
HL1_HBI_2	5.76	98.36	97.60%	43.72	0.003007	98.32	93.04
HL1_HBI_3	5.39	98.20	97.59%	43.85	0.003006	98.28	93.02
HL1_CL_1	6.70	98.24	96.45%	43.96	0.003029	98.32	93.14
HL1_CL_2	5.52	98.29	98.05%	43.71	0.003001	98.27	92.94
HL1_CL_3	5.86	98.37	97.63%	43.74	0.002966	98.30	92.99
HL2_HBI_1	5.70	98.82	97.78%	44.15	0.004281	99.03	97.18
HL2_HBI_2	6.12	98.67	97.75%	44.23	0.004253	98.92	96.91
HL2_HBI_3	7.02	98.52	97.73%	44.05	0.003249	98.81	96.61
HL2_CL_1	6.76	98.51	97.50%	43.74	0.003378	98.82	96.57
HL2_CL_2	6.94	98.54	97.82%	43.96	0.003637	98.85	96.71
HL2_CL_3	7.11	98.76	97.66%	43.92	0.003245	99.03	97.20

**Table 2:** Basic information on the poplar materials and experimentation site.

Species	Variety Name	Number	Parental	Breeding Time	Afforestation Time and Density	Afforestation Method
*P. deltoides*	*P. deltoides* ‘Jianghan 1’	HBI	*P. deltoides* ‘Zhongshi 8’ (♀)	2012	January 2015 (3 m × 10 m)	Dryland afforestation (two-year-old plant)
			*P. deltoides* ‘2-46’ (♂)			
	*P. deltoides* ‘Chulin 2’	CL	Natural hybrid progeny of *P. deltoides* ‘I-69’	2009		
Trial forest information	Geographical position	Lijiazhou Forest Farm, Huanggang City, Hubei Province (114°42′ E, 30°25′ N)				
	Environmental characteristics	Situated on the northern bank of the middle reaches of the Yangtze River, the area is characterized by low and flat terrain, with elevations of 18–35 m. The annual average temperature is 16.9 °C, accompanied by an annual precipitation of 1182.5 mm, an average relative humidity of 77%, and a frost-free period lasting 208 d. The riverbanks experience submersion for 40–70 d·year^−1^, with a maximum flooding depth of approximately 3.0 m. The soil’s parent material consists of alluvial deposits from the Yangtze River, predominantly composed of clay and sandy loam (pH = 7.4–8.5) [85].				
	Vegetation characteristics	Artificial pure forest, with a few herbaceous plants distributed beneath the canopy, mainly including *Cyperus rotundus*, *Erigeron acris*, *Carex liparocarpos*, *Paederia scandens*, *Phragmites australis*, *Arthraxon hispidus*, et al. [85].				

## Data Availability

The data presented in this study are available upon request from the corresponding author. The data are not publicly available due to ethical reasons.

## References

[1] Wang J.H., Zhou Y., Su G.Z., Song Q.Q., Lin G.F., Xing Y., Chen Q.F., Yu L.J., Su S.H., Xie R.H. (2025). MPK3- and MPK6-mediated phosphorylation of STOP1 triggers its nuclear stabilization to modulate hypoxia responses in *Arabidopsis*. Plant Cell.

[2] Vousdoukas M.I., Paprotny D., Mentaschi L., Monioudi I.N., Feyen L. (2026). Coastal flood impacts and lost ecosystem services along Europe’s outermost regions and overseas countries and territories. Nat. Commun..

[3] Guo M., Yao Y., Yin K., Tan L., Liu M., Hou J., Zhang H., Liang R., Zhang X., Yang H. (2024). ACBP4-WRKY70-*RAP2.12* module positively regulates submergence-induced hypoxia response in *Arabidopsis thaliana*. J. Integr. Plant Biol..

[4] Zhang Y., Liang T., Dong H. (2024). Melatonin enhances waterlogging tolerance of field-grown cotton through quiescence adaptation and compensatory growth strategies. Field Crop. Res..

[5] Hou M., Lu Y., Xu S., Yu D., Jiang W., Wu J., Gao D., Li X. (2026). Physiological and molecular mechanisms underlying waterlogging tolerance in Salvia miltiorrhiza: Implications for breeding stress-resilient crops. Ind. Crop. Prod..

[6] Zhou S., Yu B., Zhang Y. (2023). Global concurrent climate extremes exacerbated by anthropogenic climate change. Sci. Adv..

[7] Fan B., Liao K., Wang L.N., Shi L.L., Zhang Y., Xu L.J., Zhou Y., Li J.F., Chen Y.Q., Chen Q.F. (2023). Calcium-dependent activation of CPK12 facilitates its cytoplasm-to-nucleus translocation to potentiate plant hypoxia sensing by phosphorylating ERF-VII transcription factors. Mol. Plant.

[8] Li G., Cheng H., Qiao C., Feng J., Yan P., Yang R., Song J., Sun J., Zhao Y., Zhang Z. (2025). Root-zone oxygen supply mitigates waterlogging stress in tomato by enhancing root growth, photosynthetic performance, and antioxidant capacity. Plant Physiol. Bioch..

[9] He Y., Sun S., Zhao J., Huang Z., Peng L., Huang C., Tang Z., Huang Q., Wang Z. (2023). UDP-glucosyltransferase OsUGT75A promotes submergence tolerance during rice seed germination. Nat. Commun..

[10] Triozzi P.M., Brunello L., Novi G., Ferri G., Cardarelli F., Loreti E., Perales M., Perata P. (2024). Spatiotemporal oxygen dynamics in young leaves reveal cyclic hypoxia in plants. Mol. Plant.

[11] Renziehausen T., Frings S., Schmidt-Schippers R. (2024). ‘Against all floods’: Plant adaptation to flooding stress and combined abiotic stresses. Plant J..

[12] Toulotte J.M., Pantazopoulou C.K., Sanclemente M.A., Voesenek L.A.C.J., Sasidharan R. (2022). Water stress resilient cereal crops: Lessons from wild relatives. J. Integr. Plant Biol..

[13] Yuan L.B., Chen M.X., Wang L.N., Sasidharan R., Voesenek L.A.C.J., Xiao S. (2023). Multi-stress resilience in plants recovering from submergence. Plant Biotechnol. J..

[14] Zheng Z., Ghouri F., Ali S., Xia W., Li Z., Sun L., Shahid M.Q. (2025). Sucrose transporters and calcium oxide mitigate cadmium toxicity in rice by regulating carbohydrate metabolism, metal transporters, and oxidative stress. Environ. Chem. Ecotoxicol..

[15] Voesenek L.A.C.J., Bailey-Serres J. (2015). Flood adaptive traits and processes: An overview. New Phytol..

[16] Bailey-Serres J., Fukao T., Gibbs D.J., Holdsworth M.J., Lee S.C., Licausi F., Perata P., Voesenek L.A., van Dongen J.T. (2012). Making sense of low oxygen sensing. Trends Plant Sci..

[17] Zhao Q., Feng Y., Shao Y., Huang J., Chen Z. (2024). Response mechanism of *Cynodon dactylon* to flooding stress based on integrating metabonomics and transcriptomics analysis. Environ. Exp. Bot..

[18] Wang F., Zhou Z., Liu X., Zhu L., Guo B., Lv C., Zhu J., Chen Z.-H., Xu R. (2024). Transcriptome and metabolome analyses reveal molecular insights into waterlogging tolerance in barley. BMC Plant Biol..

[19] Zhang X., Duan W., Wang Y., Jiang Z., Li Q. (2025). MfWRKY40 positively regulates drought tolerance in Arabidopsis Thaliana by scavenging reactive oxygen species. Int. J. Mol. Sci..

[20] Pan J., Song J., Sohail H., Sharif R., Yan W., Hu Q., Qi X., Yang X., Xu X., Chen X. (2024). RNA-seq-based comparative transcriptome analysis reveals the role of *CsPrx73* in waterlogging-triggered adventitious root formation in cucumber. Hortic. Res..

[21] Pan J., Sohail H., Sharif R., Hu Q., Song J., Qi X., Chen X., Xu X. (2025). Cucumber JASMONATE ZIM-DOMAIN 8 interaction with transcription factor MYB6 impairs waterlogging-triggered adventitious rooting. Plant Physiol..

[22] Chen L.Y., Wang J.L., Cao Y., Chen H., Yuan T., Zhang W.D., Morales-Briones D.F., Zhao S.Y., Tan N.H., Huang C.H. (2026). The convergent genomic dynamism for aquatic adaptation in vascular plants. Curr. Biol..

[23] Elsey-Quirk T., Lynn A., Jacobs M.D., Diaz R., Cronin J.T., Wang L., Huang H., Justic D. (2024). Vegetation dieback in the mississippi river delta triggered by acute drought and chronic relative sea-level rise. Nat. Commun..

[24] Subedi U., Hughes K.B., Lehmann M., Chen G., Acharya S., Hannoufa A., Nguyen C.V., Singer S.D. (2025). The down-regulation of *MsWOX13-2* promotes enhanced waterlogging resilience in alfalfa. Plant J..

[25] Peláez-Vico M.Á., Tukuli A., Singh P., Mendoza-Cózatl D.G., Joshi T., Mittler R. (2023). Rapid systemic responses of *Arabidopsis* to waterlogging stress. Plant Physiol..

[26] Yang C., Liu W., Qiu M., Xie C., Lu H., Liang W., Qiu R., Li D., Li L., Yang J. (2025). A *cis*-regulatory element of the *PHYTOCHROME* A gene confers the submergence escape capacity for amphibious plants. Nat. Commun..

[27] Li Y., Shi L.C., Yang J., Qian Z.H., He Y.X., Li M.W. (2021). Physiological and transcriptional changes provide insights into the effect of root waterlogging on the aboveground part of *Pterocarya stenoptera*. Genomics.

[28] Cho H.Y., Loreti E., Shih M.C., Perata P. (2021). Energy and sugar signaling during hypoxia. New Phytol..

[29] Liang R., Tan L., Guo X., Lou S., Dan X., Han Y., Zeng C., Zhang H., Yang K., Chen L. (2025). Allelic variation in the promoter of *WRKY22* enhances humid adaptation of *Arabidopsis thaliana*. Mol. Plant.

[30] Li Q., Duncan S., Li Y., Huang S., Luo M. (2024). Decoding plant specialized metabolism: New mechanistic insights. Trends Plant Sci..

[31] Hartman S., Sasidharan R., Voesenek L.A.C.J. (2021). The role of ethylene in metabolic acclimations to low oxygen. New Phytol..

[32] Ateeq M., Zhang D., Xiao J., Zhang H., Shen X., Meng J., Yang J., Alam S.M., Kaleem M.M., Khan M.A. (2025). Decoding submergence tolerance in *Prunus persica*: Integrated transcriptomic and metabolomic acclimations of antioxidant system, cell wall dynamics, and hormonal signaling. Hortic. Adv..

[33] Lu X., Yang L., Shen L., Zhan C., Dai L., Huang L., Zhang Q., Fang Y., Ren D., Zhu L. (2025). Genome-wide association study uncovers a novel gene responsible for rice seedling submergence tolerance. Plant Biotechnol. J..

[34] Chatterjee Y., Tomar S., Mishra M., Pareek A., Singla-Pareek S.L. (2025). OsLdh7 overexpression in rice confers submergence tolerance by regulating key metabolic pathways: Anaerobic glycolysis, ethanolic fermentation and amino acid metabolism. Plant Cell Environ..

[35] Tang R., Long Q. (2025). Multi-indicator comparison in characterizing spatiotemporal patterns of water disasters and corresponding agricultural applications in the Middle-and-lower Yangtze River. Agric. Water Manag..

[36] Liu S., Ai H., Duan S., Li R., Owusu E.A., Lv L., Sun Z., Xu D., Zhang M., Zhou A. (2026). Integrated analysis of laboratory and field experiments to screen rice germplasm for submergence tolerance across various growth stages. Field Crop. Res..

[37] Miao T., Wu Z., Liu J., Ding C., Cao Z., Sun H., Yan C., Yang C. (2025). The impact of seasonal flooding on the physicochemical properties, enzyme activity, and microbial communities of *Populus* forest soil in the Yangtze River floodplain. J. Northeast For. Univ..

[38] Dun X., Qu H., Tian Y., Fang S., Xu X. (2013). Effects of thinning treatments on soil available nitrogen of the poplar plantationsin flooding land of Yangtze River. J. Nanjing For. Univ..

[39] Chen Y., Wang H., Hu W., Wang S., Wang Y., Snider J.L., Zhou Z. (2017). Combined elevated temperature and soil waterlogging stresses inhibit cell elongation by altering osmolyte composition of the developing cotton (*Gossypium hirsutum* L.) fiber. Plant Sci..

[40] Song Q., He F., Kong L., Yang J., Wang X., Zhao Z., Zhang Y., Xu C., Fan C., Luo K. (2024). The IAA17. 1/HSFA5a module enhances salt tolerance in *Populus tomentosa* by regulating flavonol biosynthesis and ROS levels in lateral roots. New Phytol..

[41] Liu N., Van den Bulcke J., Van Acker J., Liu F., Gao C., Yu J., Su X., Liu C., Huang Q. (2025). Enhancing large-diameter timber production: Evaluating poplars by genotype and spacing. Ind. Crop. Prod..

[42] Gao C., Chen C., Liu N., Liu F., Su X., Liu C., Huang Q. (2024). Genetic diversity and association analysis of traits related to water-use efficiency and nitrogen-use efficiency of *Populus deltoides* based on SSR markers. Int. J. Mol. Sci..

[43] Gao C., Liu C., Chen C., Liu N., Liu F., Su X., Huang Q. (2024). Genetic evaluation of water use efficiency and nutrient use efficiency in *Populus deltoides* Bartr. ex Marsh. seedlings in China. Plants.

[44] Chen L., Lai J., Hu X., Yang W., Zhang J., Wang X., Tan L. (2017). Effects of inoculation with arbuscular mycorrhizal fungi on photosynthetic physiology in fe-males and males of *Populus deltoides* exposed to cadmium pollution. Chin. J. Plant Ecol..

[45] Zhao X., Cheng F., Zhang K., Huang K., Ni Y., Meng X., Tang L. (2019). Root morphology of different poplar clone seedlings under waterlogging treatment. J. Nanjing For. Univ..

[46] Miao L.F., Yang F., Han C.Y., Pu Y.J., Ding Y., Zhang L.J. (2017). Sex-specific responses to winter flooding, spring waterlogging and post-flooding recovery in *Populus deltoides*. Sci. Rep..

[47] Cao F.L., Conner W.H. (1999). Selection of flood-tolerant *Populus deltoides* clones for reforestation projects in China. For. Ecol. Manag..

[48] Nielsen J.L., Rood S.B., Pearce D.W., Letts M.G., Jiskoot H. (2010). Streamside trees: Responses of male, female and hybrid cottonwoods to flooding. Tree Physiol..

[49] Rood S.B., Nielsen J.L., Shenton L., Gill K.M., Letts M.G. (2010). Effects of flooding on leaf development, transpiration, and photosynthesis in narrow leaf cottonwood, a willow-like poplar. Photosynth. Res..

[50] Letts M.G., Phelan C.A., Johnson D.R.E., Rood S.B. (2008). Seasonal photosynthetic gas exchange and leaf reflectance characteristics of male and female cottonwoods in a riparian woodland. Tree Physiol..

[51] Li J.J., Chen Y.H., Zhou Z.H., Wang Y.J., Yao X., Guo L. (2023). Research progress on mechanisms of plant adaptation to flooding stress. Plant Sci. J..

[52] Yin J., Niu L., Li Y., Song X., Ottosen C.O., Wu Z., Jiang F., Zhou R. (2023). The effects of waterlogging stress on plant morphology, leaf physiology and fruit yield in six tomato genotypes at anthesis stage. Veg. Res..

[53] Langan P., Cavel E., Henchy J., Bernád V., Ruel P., O’dea K., Yatagampitiya K., Demailly H., Gutierrez L., Negrão S. (2024). Evaluating waterlogging stress response and recovery in barley (*Hordeum vulgare* L.): An image-based phenotyping approach. Plant Methods.

[54] Sun H., Wu Z., Liu J., Miao T., Cao Z. (2020). Effects of water stress on growth and physiological properties of four poplar varieties. J. West China For. Sci..

[55] Fàbregas N., Yoshida T., Fernie A.R. (2025). Bidirectional crosstalk between plant hormone signaling and metabolism. Plant Physiol..

[56] Leeggangers H.A., Rodriguez-Granados N.Y., Macias-Honti M.G., Sasidharan R. (2023). A helping hand when drowning: The versatile role of ethylene in root flooding resilience. Environ. Exp. Bot..

[57] Hartman S., Liu Z., Van Veen H., Vicente J., Reinen E., Martopawiro S., Zhang H., Van Dongen N., Bosman F., Bassel G.W. (2019). Ethylene-mediated nitric oxide depletion pre-adapts plants to hypoxia stress. Nat. Commun..

[58] Huang K., Zhang X., Yan X., Qin M., Lv L., Yang S., Zheng Y., Yin G., Tian X., Wang X. (2026). Endothelial-targeted modification of ginseng-derived exosomes for IL-6 SiRNA delivery ameliorates hepatic ischemia-reperfusion injury. J. Nanobiotechnol..

[59] Yu W.W., Chen Q.F., Liao K., Zhou D.M., Yang Y.C., He M., Yu L.J., Guo D.Y., Xiao S., Xie R.H. (2024). The calcium-dependent protein kinase CPK16 regulates hypoxia-induced ROS production by phosphorylating the NADPH oxidase RBOHD in *Arabidopsis*. Plant Cell.

[60] Dong Y., Ma H., Shen Y., Li P., Ge C., Shen Q., Li J., Liu R., Zhang S., Liu S. (2025). Ethylene enhances cold resistance through GhDREB1/CBF in cotton (*Gossypium hirsutum* L.). Plant J..

[61] Pan R., Buitrago S., Feng X., Hu A., Zhou M., Zhang W. (2022). Ethylene regulates aerenchyma formation in cotton under hypoxia stress by inducing the accumulation of reactive oxygen species. Environ. Exp. Bot..

[62] Hao D., Li W., Guo H. (2025). Ethylene signaling in *Arabidopsis*: A journey from historical discoveries to modern insights. Plant Horm..

[63] Rankenberg T., van Veen H., Sedaghatmehr M., Liao C.Y., Devaiah M.B., Stouten E.A., Balazadeh S., Sasidharan R. (2024). Differential leaf flooding resilience in *Arabidopsis thaliana* is controlled by ethylene signaling-activated and age-dependent phosphorylation of ORESARA1. Plant Commun..

[64] Wu J., Wang J., Wang P., Su C., Hui W., Gong W. (2023). Ethylene-induced improvement in photosynthetic performance of *Zanthoxylum armatum* under reoxygenation conditions. Funct. Plant Biol..

[65] Tan X., Liu M., Du N., Zwiazek J.J. (2021). Ethylene enhances root water transport and aquaporin expression in trembling aspen (*Populus tremuloides*) exposed to root hypoxia. BMC Plant Biol..

[66] Tang H., Wang Q., Xie H., Li W. (2024). The function of secondary metabolites in resisting stresses in horticultural plants. Fruit Res..

[67] Li C., Pei J., Yan X., Cui X., Tsuruts M., Liu Y., Lian C. (2021). A poplar B-box protein PtrBBX23 modulates the accumulation of anthocyanins and proanthocyanidins in response to high light. Plant Cell Environ..

[68] Volf M., Volfová T., Hörandl E., Wagner N.D., Luntamo N., Salminen J.-P., Sedio B.E. (2022). Abiotic stress rather than biotic interactions drives contrastingtrends in chemical richness and variation in alpine willows. Funct. Ecol..

[69] Lewis D.R., Ramirez M.V., Miller N.D., Vallabhaneni P., Ray W.K., Helm R.F., Winkel B.S., Muday G.K. (2011). Auxin and ethylene induce flavonol accumulation through distinct transcriptional networks. Plant Physiol..

[70] Watkins J.M., Hechler P.J., Muday G.K. (2014). Ethylene-induced flavonol accumulation in guard cells suppresses reactive oxygen species and moderates stomatal aperture. Plant Physiol..

[71] Watkins J.M., Chapman J.M., Muday G.K. (2017). Abscisic acid-induced reactive oxygen species are modulated by flavonols to control stomata aperture. Plant Physiol..

[72] Jiang G., Wang X., Jiang Q., Bai Y., Zhang L., Zhang P., Liu J., Li L., Li H., Huang L. (2025). Mechanisms of flavonoids in quinoa’s response to flooding stress in grain filling stage. Front. Plant Sci..

[73] Wang X., Zhang P., Qin Z., Jiang G., Bai Y., Zhang L., Liu J., Li L., Li H., Huang L. (2025). Joint analysis of transcriptome and metabolome on the accumulation mechanism of flavonoids in quinoa seedlings under flooding stress. BMC Plant Biol..

[74] Wang L., Pan X., Jiang L., Chu Y., Gao S., Jiang X., Zhang Y., Chen Y., Luo S., Peng C. (2022). The biological activity mechanism of chlorogenic acid and its applications in food industry: A review. Front. Nutr..

[75] Buitrago S., Yang X., Wang L., Pan R., Zhang W. (2025). Evolutionary analysis of anthocyanin biosynthetic genes: Insights into abiotic stress adaptation. Plant Mol. Biol..

[76] Mizutani M., Ohta D., Sato R. (1997). Isolation of a cDNA and a genomic clone encoding cinnamate 4-hydroxylase from Arabidopsis and its expression manner in planta. Plant Physiol..

[77] Karlson C.K.S., Mohd Noor S.N., Khalid N., Tan B.C. (2022). CRISPRi-mediated down-regulation of the cinnamate-4-hydroxylase (C4H) gene enhances the flavonoid biosynthesis in *Nicotiana tabacum*. Biology.

[78] Strygina K., Khlestkina E. (2022). Flavonoid biosynthesis genes in *Triticum aestivum* L.: Methylation patterns in cis-regulatory regions of the duplicated *CHI* and *F3H* genes. Biomolecules.

[79] Liu X., Yang W., Mu B., Li S., Li Y., Zhou X., Zhang C., Fan Y., Chen R. (2018). Engineering of ‘*Purple Embryo* Maize’ with a multigene expression system derived from a bidirectional promoter and self-cleaving 2A peptides. Plant Biotechnol. J..

[80] Zhong R., Morrison W.H., Negrel J., Ye Z.H. (1998). Dual methylation pathways in lignin biosynthesis. Plant Cell.

[81] Lu J., Zhao H., Wei J., He Y., Shi C., Wang H., Song Y. (2004). Lignin reduction in transgenic poplars by expressing antisense *CCoAOMT* gene. Prog. Nat. Sci..

[82] Habibi F., Doron M., Jacobson T., Voiniciuc C., Brecht J.K., Sarkhosh A. (2025). Postharvest hypoxia and anoxia stresses delay anthocyanin accumulation in cold-stored blood orange fruit. Postharvest Biol. Tech..

[83] Lu X., Gu X., Shi Y. (2022). A review on lignin antioxidants: Their sources, isolations, antioxidant activities and various applications. Int. J. Biol. Macromol..

[84] Wang H., Liu S., Fan F., Yu Q., Zhang P. (2022). A moss 2-Oxoglutarate/Fe (II)-dependent dioxygenases (2-ODD) gene of flavonoids biosynthesis positively regulates plants abiotic stress tolerance. Front. Plant Sci..

[85] Li Y., Hu X., Zhou J., Yang S. (2010). Effect evaluation of *Oncomelania* control by changing environment in river beaches based on principal component analysis and clustering analysis. J. Cent. South Univ. For Technol..

[86] Wei S., Zheng B., Wang S., Yang X., Chen Y., Yin T. (2025). Integrated analysis of *Populus deltoides* PR1 genes uncovered a *PdePR1* as a defense marker against foliar rust. Plant Physiol. Biochem..

[87] Livak K.J., Schmittgen T.D. (2001). Analysis of relative gene expression data using real-time quantitative PCR and the 2(-Delta Delta C(T)) Method. Methods.

